# “I Crave a Blunt, I Don’t Crave a Cigarillo”: A Focus Group Study on Perceptions of Nicotine and Addiction among US Adults Who Currently Smoke Little Cigars or Cigarillos

**DOI:** 10.3390/ijerph20065086

**Published:** 2023-03-14

**Authors:** Emily E. Hackworth, Charity A. Ntansah, Katherine C. Henderson, Di Pei, Reed M. Reynolds, Hue Trong Duong, Bo Yang, David L. Ashley, James F. Thrasher, Lucy Popova

**Affiliations:** 1 Department of Health Promotion, Education and Behavior, Arnold School of Public Health, University of South Carolina, Columbia, SC 29208, USA; 2 School of Public Health, Georgia State University, Atlanta, GA 30302, USA; 3 Communication Department, University of Massachusetts, Boston, MA 02125, USA; 4 Department of Communication, Georgia State University, Atlanta, GA 30302, USA; 5 Department of Communication, University of Arizona, Tucson, AZ 85721, USA

**Keywords:** little cigars, cigarillos, addiction, nicotine, perceptions, qualitative, communication

## Abstract

While the US Food and Drug Administration (FDA)’s proposal to reduce the nicotine content in cigarettes is gaining traction, it is still undetermined whether the policy will also include other combustible tobacco products, such as little cigars and cigarillos (LCCs), and how such a policy should be communicated given the patterns of use and perceptions around LCCs. This study examined perceptions of nicotine and addiction related to LCC use and involved data collection from eight semi-structured virtual focus groups conducted in Summer 2021 in the US. Participants were adults who reported past-30-day use of LCCs, consisting of African American males (*n* = 9), African American females (*n* = 9), white males (*n* = 14), and white females (*n* = 11). Participants discussed their perceptions of nicotine and addiction in general and in relation to LCC use. Inductive thematic analysis of transcripts was conducted. Differences across race and sex groups were examined. Participants did not consider nicotine to be a characterizing feature of LCCs; rather, they generally associated nicotine with cigarettes. Participants’ views of nicotine and addiction related to LCCs were discussed along four dimensions: context of use, frequency of use, the presence of cravings, and whether a product is modified (e.g., by adding marijuana). Social and infrequent use, a lack of cravings, and the use of LCCs for marijuana were considered indicative of a lack of addiction and reasons not to be concerned about nicotine in LCCs. Because perceptions of nicotine and addiction related to LCCs differ from those of cigarettes, communications about a reduced nicotine policy that includes LCCs should consider these differences to ensure the policy is understood by people who currently use LCCs and to prevent people who use cigarettes from switching to LCCs.

## 1. Introduction

The United States Food and Drug Administration (FDA) announced in June of 2022 that plans to implement a reduced-nicotine standard for combustible cigarettes will move forward [1]. By reducing the nicotine content of cigarettes—and therefore their addictiveness—this policy aims to reduce smoking rates, make smoking cessation easier, and prevent tobacco initiation among youth and young adults [2]. The FDA is considering how broadly this policy should be implemented, as focusing solely on cigarettes may encourage people who smoke cigarettes to shift to other combustible tobacco products or engage in the dual use of cigarettes and other products, reducing the policy’s effectiveness [3]. Therefore, there are sound public health reasons to include other combustible tobacco products in the policy.

In the US, cigars, including both large cigars and little cigars and cigarillos (LCCs), are the third most used tobacco products after cigarettes and e-cigarettes. In 2020, 12.5% of US adults used cigarettes and 3.5% used cigars [4]. Rates of cigarette and cigar use are similar in Canada [5]. In the UK, cigar use is less common; however, a number of people who smoke report concurrent cigar use [6]. Amid the declining trend of cigarette consumption in recent years, cigar consumption has increased, with rates more than doubling from 2000 to 2011 [7]. Recent years also have witnessed an increase in cigar promotion on social media, which might lead to more LCC consumption [8].

Smoking LCCs puts individuals at risk for the same adverse health effects as smoking cigarettes, including cancer, lung disease, heart disease, and gum disease [9]. Indeed, smoking LCCs can deliver more tobacco-specific nitrosamines, carbon monoxide, and nitrogen oxide than smoking a cigarette due to their larger size [10]. Furthermore, nicotine delivery from LCCs is similar to cigarettes [11]. Despite their similarities, cigar products are taxed at lower rates than cigarettes in both the US and UK, resulting in lower prices [12,13]. In the US, weaker advertising restrictions apply to products labeled as cigars than to cigarettes [14]. Therefore, while some cigars look similar to cigarettes, they meet the legal definition of cigars, and tobacco manufacturers label them as LCCs to avoid the more stringent regulations of cigarettes and exploit the policy [12,14,15].

There are several important distinctions between the patterns of cigarette and LCC use. While cigarette smoking rates are similar between US whites and African Americans, cigar use prevalence is much higher among African Americans [16]. Additionally, males and people with lower incomes are more likely to use LCCs than their counterparts [17]. Another distinction between cigarette and cigar use concerns the patterns and contexts of use. Overall, LCCs are commonly used in infrequent social occasions rather than daily [18]. LCCs also come in many flavors that are banned in cigarettes [19], and flavors are a primary reason people cite for using LCCs [20]. Many LCCs are designed to be modified by the user, such as emptying out the tobacco and using the LCC wrapper to hold marijuana, which is very common [21].

As the tobacco industry has successfully marketed LCCs as different from cigarettes, they have also succeeded in fostering misperceptions. People falsely believe that cigars are less addictive than cigarettes [17], and many people who use LCCs do not consider themselves addicted [20]. If a reduced nicotine policy was implemented for LCCs and the initiative was communicated to the public, misbeliefs about LCCs might reduce public understanding about the policy. Research shows that anti-smoking communication campaigns that target people who use LCCs should be differentiated from anti-smoking campaigns that target people who use cigarettes. For example, a study aiming to develop a health communication campaign to discourage cigarillo use found that given that individuals who use these products tend to be younger and misperceive them to be less harmful than traditional cigarettes, messages targeting people who use cigarillos should correct those misperceptions and focus on the harmful constituents of LCCs [22]. To help understand the potential for reduced nicotine LCCs to lower the population harm from the use of these products and inform communication efforts about the policy, we conducted a qualitative study of people who use LCCs to provide an in-depth examination of their perceptions of nicotine and addiction related to LCC use.

## 2. Materials and Methods

In the US, the prevalence of LCC use is highest among non-Latinx African American males (AAM) (12%), non-Latinx African American males (8%), non-Latinx African American females (AAF) (4%), and non-Latinx white females (WF) (2%) [16].

To capture the perspectives of these four populations, we conducted two separate focus groups for each population (*n* = 8 focus groups) recruited from around the US by a marketing research company. Eligible participants: (1) were 18–44 years old, (2) used LCCs in the past 30 days, (3) identified as non-Latinx white or non-Latinx African American, and (4) had access to sufficient technology to participate in virtual focus groups. In total, 975 people were screened for eligibility, 76 eligible participants were scheduled, and 43 took part in one of 8 focus groups.

All focus groups were conducted using Adobe Connect. Each group included three to eight participants and lasted 75–90 min. Participants were compensated with USD 100 for their time. At the beginning of the focus groups, all participants provided informed consent. The study was approved by the University of South Carolina’s IRB.

The moderator guide was based on our previous work [23,24], with adaptations specific to people who use LCCs. All focus groups began with open-ended questions about perceptions about nicotine and addiction as they relate to LCCs, the health effects of LCCs, and participants’ experiences with LCCs. The second part of the focus groups involved showing participants a series of messages communicating the reduced-nicotine policy. Reactions to these messages are reported elsewhere [25]. In this paper, we focus on participants’ perceptions of nicotine and addiction related to LCCs that were collected before message exposure.

Transcripts were reviewed by our team for accuracy, uploaded into NVivo version 12.0, and analyzed using inductive thematic analysis. The preliminary codebook was created by the first two authors, who reviewed the transcripts and recorded prominent themes. Then, the codebook was shared with all authors, including multiple content experts, and revisions were made based on their feedback. The first two authors then randomly selected two transcripts to independently code and then reviewed the coding with the last author. Any coding discrepancies were discussed, and code definitions were adjusted as necessary to create the master codebook. The first two authors then randomly divided the remaining transcripts and coded them independently. Once coding was complete, all authors received narrative segments of specific codes and wrote memos for their assigned codes. After a group discussion, the first author then reviewed all memos and synthesized the results.

## 3. Results

### 3.1. Participant Characteristics

Participant characteristics are shown in Table 1. The sample was 53% male, 56% white, and 49% 18–29 years old; 65% reported concurrent use of LCCs and cigarettes, and 61% reported current marijuana use.

### 3.2. Nicotine

#### 3.2.1. Free Associations with Nicotine

When asked: “what is the first thing that comes to mind when you hear the word ‘nicotine’?”, many participants mentioned positive outcomes associated with nicotine, regardless of race or sex. They described positive psychoactive effects including “relaxation”, “happy time”, and “a stress reliever”. One participant described a “peaceful feeling when you get that nicotine buzz” (WF). Some participants recognized the downfall of this positive feeling, as one participant commented that nicotine “simulates a happiness in you. However, you kinda grow weaker to it the more you do it, but it becomes the only way you could get happy” (AAM). Alternatively, this association was less positive for participants in other groups. For example, some participants mentioned the direct link between nicotine and addiction. Participants in a few focus groups also associated nicotine with specific brands of cigarettes and e-cigarettes, nicotine replacement therapy products (i.e., the nicotine patch), or “tobacco”.

#### 3.2.2. Perceptions of Nicotine in LCCs

Across all eight focus groups, at least some participants had not previously thought about LCCs containing nicotine. Some participants indicated that they “didn’t know there was nicotine in cigarillos” (AAM). In addition, how participants viewed the nicotine in LCCs was related to their frequency of use and the context or situations in which they used them. When discussing their use of LCCs, many reported non-daily LCC use. For this reason, these participants were not concerned about the amount of nicotine in LCCs because they deemed them as something they did not frequently use. All focus groups had some participants who described their use of LCCs as only occurring in certain circumstances, such as social events or special occasions, when they used LCCs as a “treat”. One participant said, “I’m not using it for the nicotine. It’s more for that social aspect” (WF). These participants felt that nicotine was not a key feature of LCCs because they used them situationally. Participants in several groups stated that because they did not experience cravings for LCCs, the nicotine level in LCCs must be low.

Many participants, particularly those in the AAM and AAF groups, described their modification of LCCs for “blunts” with marijuana. For example, participants said that they “mix it half and half” (AAM), or “take all the tobacco out and replace it with marijuana” (AAF). When participants were asked about where the nicotine was located in an LCC, some were unsure, whereas others responded that nicotine was in both the tobacco filler and the wrapper. Other participants who used only the wrappers said that they were not concerned about the nicotine content of LCCs because nicotine was only in the tobacco leaf filler and the wrappers just “hold it together”. For example, one participant said, “most people who buy cigarillos is usually just for the paper, not even necessarily the nicotine or tobacco” (AAM).

In general, participants reported a perceived low frequency of use, a social context of use, no experience of cravings, and LCC modification for blunts as evidence that they consumed little nicotine when using LCCs. In only a few groups did some participants indicate that nicotine was a concern. For instance, one participant acknowledged that “anything that’s a tobacco product has some sort of nicotine” (AAM). In addition, some of these participants commented that the combination of nicotine and marijuana had a unique effect. One participant said, “When I started smoking weed, it was originally just weed. Once I started mixing tobacco, I don’t want to smoke just weed now. It needs to have the tobacco and that’s probably because of the nicotine” (AAM).

#### 3.2.3. Nicotine in LCCs Compared with Cigarettes

When asked to compare the amount of nicotine in LCCs to cigarettes, participants across focus groups could not reach consensus. Some participants in each group felt that there was more nicotine in cigarettes because they experienced “more of satisfaction from cigarettes” (AAM). Other participants believed LCCs contained more nicotine because “they are harder to inhale than cigarettes” (AAF) and that they were “more potent”. Some participants also said that they had heard “when you smoke a cigar, it’s like smoking 10 cigarettes at once” (WM), but they were not certain about this comparison. Some participants focused on the behaviors of smoking cigars versus cigarettes to suggest a difference in the amount of nicotine intake. For example, they described “puffing” on LCCs and “inhaling” cigarettes, which they argued led to different amounts of nicotine ingestion.

#### 3.2.4. Health Effects and Harshness of Nicotine in LCCs

When asked about the health effects of nicotine in LCCs, most groups listed the health effects of combusted tobacco products, including “cancer”, “lung and heart disease”, and “gum disease”. In nearly every group, however, participants also had mixed perceptions about whether nicotine directly causes such health outcomes. For example, one participant responded, “I don’t think nicotine causes any diseases. I think that’s the carcinogens. I think besides addiction, I don’t think it actually causes the diseases” (WM). Another participant said, “I’m not a doctor, but I wouldn’t consider the nicotine itself to be the cause of most diseases” (WM). Interestingly, some participants described beneficial effects of LCC use as an “appetite suppressant” (WM) and stress reliever, for example, that “they have helped me cope with hard times” (AAF).

Similar to discussions about nicotine in LCCs, participants tended to compare the health effects of LCCs with cigarettes. “Heavy” and “harsh” were words used to characterize the relative harm of these products; however, there was no consensus about which product was “heavier” or “harsher”. One participant said that “LCCs are the heaviest, the cigarettes are a little light” (AAM). Another said, LCCs “are a lot harsher” (WM). Alternatively, other participants felt that LCCs are “really smooth, not harsh like a cigarette” (AAM), and that cigarettes “feel harsher” and feel as though they are “too much” in comparison with LCCs. Another participant stated that LCCs were less harmful than e-cigarettes because they had never “heard of anybody that went to hospital and now they’re on oxygen because they can’t breathe on their own because they smoke a cigarillo” (AAF).

### 3.3. Addiction

#### 3.3.1. Free Associations with Addiction

When asked what they think about when they hear the word “addiction”, all groups discussed substances people could be addicted to, the effects of withdrawal, and that an addiction is something that “interferes with day-to-day life” (AAF). One participant said, “it starts with the physical craving, but then you get mentally obsessed with whatever substance you’re using” (WM). Others described addiction as a “weakness” and that it causes people to “be less than yourself” and have a “lack of control”. Another participant proposed that “if you don’t need it to stay alive and you can’t go three days without it, it’s probably an addiction” (AAM). Several participants identified that addiction is a disease with the potential to become deadly and that it can cause anxiety and shakes. In only one group was nicotine mentioned in association with addiction.

#### 3.3.2. Perceptions of Addiction to LCCs

When asked to describe what it means to be addicted to LCCs, participants in all groups struggled to acknowledge any connections between addiction and the use of LCCs. One participant said they “don’t think anyone’s addicted to cigarillos” (AAF). Some referred to LCCs as a “special treat” rather than an addictive product since their flavor and smell brought about a joyful feeling. Participants would consider someone addicted to LCCs if they “spend a lot on it”, “can’t stop”, “smoke them every day”, or experience “urges” to smoke LCCs. Another participant said, “addiction is typically coupled with people hiding their behavior because they start to feel shame about it” (WM).

Most participants in the groups agreed that they were not addicted to LCCs. Similar to their explanations for not being concerned about the nicotine content in LCCs, participants discussed how frequency of use, context of use, experience with urges or cravings, and the modification of LCCs would determine whether they were addicted. Many participants cited their infrequent use as evidence for their lack of addiction. Several participants explained how they “can go for a couple of days without having one”, and that if they were addicted to LCCs, they would not be able to do this. Other participants suggested that a person was considered addicted if they used LCCs daily. Several participants agreed with this suggestion because they do not experience cravings or urges to smoke LCCs and, therefore, they believed that they must not be addicted to them. A few participants said they were not addicted to LCCs because it was just a “habit”. One participant said they could “wait it out”, indicating that they were not addicted to using LCCs.

Many participants referred to the circumstances of their use of LCCs as indicative of a lack of addiction. Because they only “do it socially” and at special events, they are not addicted to LCCs. Some participants felt that even though they chose to use LCC paper as opposed to wrapping paper in their blunts, they were addicted to marijuana and not nicotine, with one participant saying, “I crave smoking a blunt, I don’t crave a cigarillo” (AAM). Others agreed that because they were only using the wrapper, which they believed does not contain nicotine, they are not addicted to LCCs.

However, there were some who acknowledged their addiction to LCCs. The AAM groups generally agreed that they were addicted to LCCs and that they were “waiting for this (focus group) to finish so they can go smoke” (AAM). One participant referred to their addiction to LCCs as “functioning” because “nothing in my life is compromised by this” (AAM). Meanwhile, participants in two focus groups (WF, AAF) were unsure about whether they were addicted to LCCs because they could “go without it” if they had to but do “like to smoke them a lot”.

#### 3.3.3. Addiction to LCCs Compared with Cigarettes and Other Products

Since many participants in all groups reported either current or former cigarette smoking, comparisons between addiction to the products were abundant. Participants in all groups compared the frequency of their use of the products as being indicative of addiction to one product and not the other. Some participants mentioned using cigarettes much more frequently than LCCs. Other participants mentioned chain smoking suggests addiction and is more plausible with cigarettes. For example, one participant said, “if you want to chain smoke… with cigars it’s not so easy because they’re physically a lot harsher than cigarettes” (AAM). A few participants admitted that they were unsure which product they were addicted to, if any, because they “smoke them equally” (AAF). One participant equated addiction to cigarettes with addiction to LCCs: “If you smoke a lot of cigars, it’s not really that different from cigarettes... it’s still tobacco. It’s still nicotine” (AAM).

Several groups discussed how the social use of LCCs impacts its addictiveness compared with cigarettes. For example, one participant said, “it’s more socially acceptable to smoke two or three cigarettes versus two Black and Milds” (AAF). Another said, “cigarettes are something you do on a daily basis… cigarillos are something that’s social, on the weekends” (WM). Another participant in the same group responded, “I feel like the cigarillo companies kind of know that because they sell their cigarillos in packs of two, whereas cigarettes, you’re getting 20 at a pack” (WM).

In contrast to how participants mostly discussed that they were not addicted to LCCs, many admitted to being addicted to cigarettes, citing their experience with cravings and withdrawal. One participant said he was not addicted to LCCs because a “cigarette satisfies a nicotine craving more” (AAM). Several participants emphasized that they believed they were addicted to cigarettes but not LCCs because they could go without LCCs. For example, one participant said, “I could go for months without a little cigar if it came to that, whereas when I was smoking cigarettes, if I had to go an hour, I was gonna kill someone” (WF). Another said, “whenever I’m stressed out, I always think to myself, boy, I need a cigarette. I never think that with a cigarillo or any other tobacco product” (WM).

While cigarettes were the dominant comparison, WM groups discussed the addictiveness of LCCs compared with other products aside from cigarettes. One participant felt that LCCs were less addictive than dip, which “was a lot more difficult to stop using” (WM) than any other tobacco product. Both WM groups discussed alcoholism and similarly felt that addiction to alcohol was much more significant than addiction to LCCs. For example, one participant said, “when I think of addiction, my family and how alcoholism impacted them and me. That’s very different from going out with a few friends every once in a while, and smoking a blunt” (WM). Another participant mentioned just getting out of rehab and clearly differentiated their addiction to alcohol from their lack of addiction to LCCs.

## 4. Discussion

To inform potential communication efforts about reduced nicotine policy for combustible products, this study examined perceptions of nicotine and addiction among people who use LCCs. Despite their current use of LCCs, most participants were not concerned about the nicotine content or addictive potential of LCCs. Previous studies have found similar perceptions, including the relatively high proportion of people who use cigars who do not consider themselves addicted [20]. In our study, participants characterized the addictiveness of LCCs, or lack thereof, through four dimensions: the frequency of use, context of use, presence of cravings, and modification of the LCC product.

Consistent with other research [26], many participants did not consider themselves addicted because they do not smoke LCCs frequently enough. Only a few participants, who smoked LCCs daily, believed they were addicted to LCCs and felt that their daily use was indicative of addiction. This viewpoint fits with the broader concept of addiction, since frequency of smoking or cigarettes smoked per day is a key indicator of dependence on cigarettes [27].

Our study showed that people who use LCCs believed that the context in which they use LCCs supports their lack of addiction to LCCs. Because participants used LCCs most frequently in social settings, and not throughout a typical day, they believed that they controlled the amount of LCC consumption rather than being addicted to using the product. Furthermore, one participant suggested that addiction involves social stigma (i.e., people feel shame and try to hide an addictive behavior), while using LCCs was viewed as socially acceptable. Indeed, the social use of LCCs is related to positive attitudes toward LCCs [26] and explains why many people use these products [18,28]. This is consistent with our findings.

Experiencing cravings is a primary component of nicotine dependence measures, such as the Wisconsin Inventory of Smoking Dependence Motives (WISDM) [29] and others, including the Nicotine Dependence Syndrome Scale (NDSS) [30], the Fagerstrom Test for Nicotine Dependence (FTND), and the Heaviness of Smoking Index (HSI) [31]. Participants in this study expressed that their lack of cravings for LCCs meant they were not addicted. They reported only using LCCs when they chose to do so, rather than in response to a craving or symptoms of withdrawal.

Product modification with marijuana was also a recurring component of participants’ perspectives of addiction to LCCs. Participants were unsure if the nicotine was predominantly in the filler, wrapper, or all parts of the product. Participants’ belief that modifying LCCs changes their addictiveness mirrors results of other studies, which found that people who use LCCs with marijuana or modify LCCs perceive the modified products as less harmful and/or less addictive than unaltered LCCs [32,33,34,35].

Participants in all groups conflated the health effects of nicotine with the health effects of tobacco combustion, which is in line with extensive research showing that people believe that nicotine is the main cause of tobacco-related diseases [36,37,38]. However, similar to our previous study with people who currently or formerly smoke cigarettes [24], at least one participant in each group attempted to correct other participants, accurately describing how nicotine is the addictive chemical in LCCs but that it does not cause health effects known to be caused by tobacco products.

The only topic that differed across race or sex groups was perceived addiction to LCCs. AAMs consistently expressed the belief that they were addicted to LCCs, whereas participants in other groups mostly stated that they were not. WMs cited experience with addiction to other tobacco products and alcohol as proof that they had experienced addiction, but not addiction to LCCs. These findings are different from what has been previously reported. One nationally representative study found that, across racial groups, females were more likely to consider themselves addicted to LCCs than males [20]. Our use of purposive sampling may have resulted in findings that differ from what would be found among the general population.

### 4.1. Implications for Research

More research is needed on the role of nicotine in LCCs use. While our participants mostly dismissed nicotine as the reason for using LCCs or LCC wrappers, it is unclear why they would use LCC paper as opposed to regular wrapping paper since LCC paper would be more expensive and take more time to modify. In examining the role of nicotine in LCC use, it might be useful to consider the distinction between the two roles nicotine plays. Studies have identified two dominant types of smoking dependence based on neural mechanisms of nicotine absorption: “peak seekers”, who smoke cigarettes to reach a peak amount of nicotine in their blood (positive reinforcement), and “trough maintainers”, who smoke more frequently to maintain a constant amount of nicotine in their blood (to avoid negative reinforcement) [39,40,41]. These types of smoking dependence are associated with race, where African American people who smoke are more likely to be “peak seekers” and white people who smoke are more likely to be “trough maintainers”. Future studies should explore how these physiological mechanisms are related to the perceptions and use of LCCs.

Our study suggests that LCC use and dependence may be better understood through the development of a nicotine dependence measurement tools specific to LCC use. While the measurement of addiction to cigarettes or cigarette-related nicotine dependence is well established [29,42,43,44], our study suggests that quantitative measures of addiction to LCCs may require new approaches. Our participants described their LCC use as resulting in positive effects (e.g., to relax, to feel good) as opposed to relieving the negative feelings (e.g., satisfy cravings) generally described among people who are addicted to cigarettes [24,30]. Future research may consider these positive-effect domains as they relate to LCCs.

### 4.2. Implications for Communication and Regulation

If the FDA’s proposed nicotine reduction policy is expanded to include LCCs, our study provides evidence that communicating about this policy with people who use LCCs may be a challenge. Participants believed that they consume little to no nicotine when using LCCs, and they did not consider themselves addicted. Product modification for smoking marijuana was also very common and reinforced beliefs that LCCs were not addictive. Therefore, people who use LCCs appear likely to believe that reducing nicotine levels in these products will not make a difference. However, previous research suggests that blunt use is associated with increased risks of addiction to both nicotine and cannabis [45]. Moreover, some participants perceived LCC use as a normative social behavior that others accept. This perceived injunctive norm (i.e., perceptions of social approval of a behavior [46]) may motivate people to engage in this behavior [47]. Communication efforts are needed to correct these misperceptions to help people better understand the reduced nicotine policy should it include LCCs [48]. Efforts to communicate about a policy to reduce nicotine in combustible tobacco products may need to frame the problem in a different way for LCCs than for cigarettes. People who use LCCs may first need to be informed of the impact of nicotine on their continued use of a product that delivers harmful chemicals and results in a wide range of adverse health effects.

As research examines communication about reduced nicotine in cigarettes, it is important to understand the impacts of this communication on people’s motivations and behaviors about switching to LCCs, particularly if the policy only includes cigarettes and LCCs become a more viable alternative for nicotine delivery.

### 4.3. Limitations

Because of the COVID-19 pandemic, focus groups were conducted virtually, which may have limited participation from individuals without internet access and video-call capability. However, virtual discussion allows participation from a wide range of geographic locations. Furthermore, there is evidence that participants’ comfort in their own homes improves the quality of their responses [49].

Our recruitment strategy involved purposive sampling; however, because we recruited specific race and sex combinations, our procedures helped assess and compare perceptions among these specific groups. Given that marijuana was a frequent topic within our focus groups, not all participants may have been willing to disclose marijuana use. While our results suggest race and sex differences, due to our purposive sampling, these differences should be examined with a larger, more representative sample.

## 5. Conclusions

Participants’ conceptualizations of nicotine in and addiction to LCCs involved four dimensions: frequency of use, context of use, the presence of cravings, and whether a product is modified. Because they used them infrequently, in social settings, did not crave them, and often replaced the tobacco filling with marijuana, most participants in our focus groups did not consider themselves to be addicted to LCCs. However, some in the AAM groups believed they were addicted to LCCs. These findings suggest that communication of a reduced nicotine policy should carefully consider the nuances of LCC use characteristics in message framing. Communication should aim to first inform people who use LCCs of the impact of nicotine and the health effects of LCCs. Message development will benefit from the use of a measurement of addiction or dependence related to nicotine in LCCs to ensure that messages are effective. To promote the reduced nicotine policy, strategies are needed to better communicate the purpose of the policy with people who currently use LCCs.

## Figures and Tables

**Table 1:** Participant characteristics.

	Total Sample (*n* = 43) *n* (%)
Sex and Race/Ethnicity (%)	
Non-Latinx white males (WM)	14 (33%)
Non-Latinx white females (WF)	11(26%)
Non-Latinx African American males (AAM)	9 (21%)
Non-Latinx African American females (AAF)	9 (21%)
Education (%)	
Less than high school	0 (0%)
High school	8 (19%)
Some college	13 (30%)
College graduate	20 (46%)
Graduate degree	2 (5%)
Age (years)	
18–29	21 (49%)
30–44	22 (51%)
# of days of LCC use in past month (%) *	
10 days or less	27 (63%)
11–29 days	6 (14%)
30 days	8 (19%)
Mean # of days of LCC use in past month	11.2 days (range: 1–30)
Predominant type of LCC use (%)	
With tobacco	16 (37%)
With marijuana	11 (26%)
With both marijuana and tobacco	16 (37%)
Past 30-day use (%)	
Cigarettes	28 (65%)
Marijuana	26 (61%)
E-cigarettes	25 (58%)
Large cigars	18 (42%)
Hookah	15 (35%)
Smokeless tobacco	9 (21%)
Heated tobacco products	3 (7%)

* Numbers do not add up to 100% due to missing data (*n* = 2).

## Data Availability

The data presented in this study are available on request from the corresponding author.
